# Protective Effect of Pycnogenol against Methotrexate-Induced Hepatic, Renal, and Cardiac Toxicity: An In Vivo Study

**DOI:** 10.3390/ph15060674

**Published:** 2022-05-27

**Authors:** Faten Al-Abkal, Basel A. Abdel-Wahab, Hanaa F. Abd El-Kareem, Yasser M. Moustafa, Dina M. Khodeer

**Affiliations:** 1 Department of Pharmacology & Toxicology, Faculty of Pharmacy, Suez Canal University, Ismailia 41522, Egypt; fatenalabkal@gmail.com (F.A.-A.); yasser_mostafa@pharm.suez.edu.eg (Y.M.M.); 2 Department of Medical Pharmacology, College of Medicine, Assiut University, Assiut 7111, Egypt; basel_post@msn.com; 3 Zoology Department, Faculty of Science, Ain Shams University, Abbasseya, Cairo 11566, Egypt; hanaafathy@sci.asu.edu.eg; 4 Department of Pharmacology & Toxicology, Faculty of Pharmacy, Badr University, Cairo 11829, Egypt

**Keywords:** cancer, methotrexate, toxicity, natural herbs, polyphenol, pycnogenol, MAPK, JNK, apoptosis, inflammation, molecular docking

## Abstract

Methotrexate (MTX) is one of the most commonly used chemotherapies for various types of cancer, including leukemia, breast cancer, hepatocarcinoma, and gastric cancers. However, the efficacy of MTX is frequently limited by serious side effects. Several studies have reported that the cytotoxic effect of MTX is not limited to cancer cells but can also affect normal tissues, leading to prospective damage to many organs. In the present study, we extensively investigated the molecular and microscopic basis of MTX-induced toxicity in different organs (liver, kidney, and heart) and explored the possible protective effect of pycnogenol, a polyphenolic component extracted from the bark of *P. pinaster*, to attenuate these effects. Biochemical analysis revealed that administration of MTX significantly reduced the function of the liver, kidney, and heart. Histological and immunohistochemical analysis indicated that MTX treatment caused damage to tissues of different organs. Interestingly, administration of pycnogenol (10, 20, and 30 mg/kg) significantly attenuated the deterioration effects of MTX on different organs in a dose-dependent manner, as demonstrated by biochemical and histological analysis. Our results reveal that pycnogenol successfully ameliorated oxidative damage and reduced toxicity, inflammatory response, and histological markers induced by methotrexate treatment. Taken together, this study provides solid evidence for the pharmacological application of pycnogenol to attenuate damage to different organs induced by MTX treatment.

## 1. Introduction

With more than 18.1 million new cases and 9.6 million deaths in 2018, cancer is considered one of the most common diseases that cause high mortality worldwide [1,2]. Several studies have been conducted to control cancer development with chemotherapeutic drugs with various modes of action. Most of the currently available chemotherapeutic drugs target the division of cancer cells, preventing tumor growth. Because they are more mitotically active than normal cells, cancer cells are more susceptible to destruction [3]. Nevertheless, chemotherapeutic drugs can also cause damage to some normal cells. Accordingly, researchers have made substantial efforts to enhance the anticancer effect of chemotherapeutic agents while also protecting normal cells against their toxicities [4]. 

**Figure 1:**
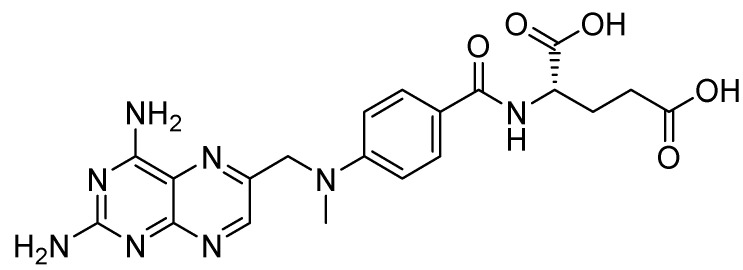
Chemical structure of methotrexate.

Among chemotherapeutic drugs, methotrexate (MTX, 2,4-diamino-N10-methyl propyl glutamic acid) is one of the most valuable and widely used for different types of cancer, such as leukemia, lymphoma, breast cancer, hepatocarcinoma, osteosarcoma, and gastric cancers (Figure 1) [3,5,6,7]. Several studies reported that MTX has a considerable potency as an antifolate, anticancer, immunosuppressive, anti-inflammatory, and common antirheumatic drug [5,8]. MTX is also used, along with other medications, to improve rheumatoid arthritis treatment [9,10,11]. The anticarcinogenic effects of MTX have been attributed to its ability to induce apoptosis by targeting caspase-dependent and caspase-independent cascades to trigger a significant stimulation of Fas and Fas-L expression to cause DNA fragmentation in carcinogenesis cells [12,13,14]. Furthermore, MTX activates nuclear factor-*κ*B (NF-*κ*B) and p38 pathways, which regulate cell apoptosis [15].

However, the efficacy of MTX is frequently limited by serious side effects. The cytotoxic effect of MTX is not limited to cancer cells; it can also affect normal tissues, leading to a high proliferation rate [3,16,17]. MTX treatment enhances the production of free radicals and causes oxidative stress, accelerating cellular damage, which can lead to inflammation, necrosis, and fibrosis in different tissues [18]. In addition, MTX has prospective side effects on many organs, such as the liver, kidney, and heart [8]. In the liver, MTX is converted to 7-hydroxymethotrexate (7-OH-MTX), the main extracellular metabolite that accumulates in a polyglutamate form in the cell [19,20,21]. When the level of polyglutamate rises, the amount of folic acid reduces, leading to hepatocyte necrosis, the main reason for MTX-induced hepatotoxicity [22,23]. MTX can also inhibit dihydrofolate reductase enzyme, which leads to impairment of liver cell membrane integrity and increases the activities of serum liver enzymes (alanine aminotransferase (ALT), aspartate aminotransferase (AST) and alkaline phosphatase (ALP)) [18,24]. 

In the kidney, MTX is precipitated in the renal tubules, which causes a reduction in the rate of glomerular filtration, as well as increased serum urea, creatinine, and uric acid levels, leading to renal failure and nephrotoxicity [8,23,24]. In the heart, MTX stimulates noticeable impairment in cardiac tissue and increases the activities of serum cardiac enzymes (creatinine kinase (CK) and CK-MB) [15]. Furthermore, MTX generates free oxygen radicals (ROS), which initiates lipid peroxidation and increases the malondialdehyde (MDA) level, leading to damage to the cell membrane [8,17,23]. It has also been reported that MTX induces tissue damage and impairment of mitochondrial function, which are associated with oxidative stress, leading to a reduction in the enzymatic antioxidant system (catalase (CAT) and superoxide dismutase (SOD) enzymes, as well as the non-enzymatic antioxidant system (reduced glutathione (GSH) level)) [8,19,20,25,26]. Furthermore, MTX shows the ability to induce nitrative stress by increasing reactive nitrogen species that initiate lipid peroxidation, damage of DNA, increase in transcription factors (nuclear factor kappa B (NF-κB)), and stimulation of inflammatory response (e.g., IL-1, IL-6, TNF-α, TGF-β, etc.) [15]. Taken together, these findings suggest the synergistic application of other medications in combination with MTX that could decrease the undesirable toxicity and side effects of MTX [27,28].

Natural herbs have been widely used as traditional medicine for many decades due to their high potency against various diseases and few side effects [29]. Natural phenolic compounds, which are prevalent in almost all meals, are natural phytochemical antioxidants with various protective benefits against several diseases, such as cancer and diabetes, as well as cardiovascular and neurological disorders [30]. Pycnogenol, a phenolic compound, represents a nutritive complement that is used as bioactive phytochemical medication all over the world. Pycnogenol was firstly used as a scientific term for this polyphenols class; however, it currently describes the particular mixture of procyanidins extracted from pine tree bark in France (*Pinus pinaster*) [31]. The obtained extract product is a fine, reddish-brown powder that is water-soluble. The consistent extract of pycnogenol is composed of phenolic components from monomers (taxifolin, epicatechin, and catechin), condensed flavonoids (grouped as procyanidins and proanthocyanidins), and phenolic acids (cinnamic acids and some glycosides). It has a protective effect against inflammatory diseases, hypertension, diabetes, and obesity, among others [32]. Furthermore, it has a beneficial effect on lung fibrosis and improves cognitive ability and cardiovascular health [33,34,35]. Pycnogenol has been recognized as a safe extracted bioactive compound according to scientific safety and preclinical toxicology records [36]. After ingestion of pycnogenol, the phenolic constituents are metabolized by the action of microbial enzymes to afford small bioactive byproducts that can be absorbed into the blood and transferred to tissues and organs [34]. These byproducts are catechin, caffeic acid, ferulic acid, taxifolin, M1 (δ-(3,4-dihydroxy-phenyl)-γ-valerolactone), and M2 (δ-(3-Methoxy-4-hydroxy-phenyl)-γ-valerolactone) [37]. M1 is considered the most active metabolite that causes anti-inflammatory impacts by inhibiting the synthesis of inducible nitric oxide synthase (iNOS) [38]. These facts indicate that pycnogenol could be synergistically used as a natural supplement to reduce the toxicity associated with MTO treatment. 

Encouraged by the aforementioned facts, in our present report, we aimed to investigating the biomolecular and microscopic bases of methotrexate-induced toxicities in different organs. Furthermore, we explored the possible protective effects of pycnogenol against methotrexate-induced toxicity through biochemical, immunohistochemical, histopathological, and molecular docking studies. 

## 2. Results

### 2.1. Biochemical Analysis

(a) Effect of MTX and pycnogenol on serum biomarkers:

i—Assessment of liver function:

First, we investigated the effect of MTX on liver function by evaluating the activity of ALT and AST enzymes (U/L) in the serum. As shown in Table 1, MTX treatment caused a significant increase at *p* < 0.05 in the activity of both ALT and AST enzymes (U/L) compared with the normal group. These results reveal that MTX caused damage to the hepatic cells, which led to the upregulation of these enzymes and enhanced their activities in serum. Next, we explored the protective effect of pycnogenol with different concentrations (10, 20, and 30 mg/kg) on serum liver function before MTX treatment. Interestingly, administration of pycnogenol led to a significant and dose-dependent decrease at *p* < 0.05 in the activity of ALT and AST enzymes (U/L), with 30 mg/kg as the most effective dose compared with the MTX group. These results indicate that pycnogenol has a protective effect against the hepatotoxicity of MTX and could maintain the integrity of the liver cells by mediating the activity ALT and AST enzymes (Figure S1). 

ii—Assessment of kidney function:

Toward the evaluation of MTX effect on kidney function, we assessed the levels of urea and creatinine (mg/dL) in the serum. As shown in Table 1, MTX treatment demonstrated a significant increase at *p* < 0.05 in serum urea and creatinine compared with the normal group. These results imply that treatment of MTX caused renal failure, which led to an increase in the level of both serum urea and creatinine (mg/dL). On the other hand, administration of pycnogenol at different concentrations (10, 20, and 30 mg/kg) before MTX treatment significantly improved serum kidney function. As shown in Table 1, pycnogenol treatment showed a significant (*p* < 0.05) dose-dependent decrease in the serum urea and creatinine levels (mg/dL), with 30 mg/kg dose as the most effective. These results indicate that pycnogenol treatment has a protective effect against MTX-induced toxicity in the kidney (Figure S1). 

iii—Assessment of heart function:

Concerning the effect of MTX on heart tissues, we evaluated the activity of heart enzymes (CK and CK-MB) (U/L) and the troponin levels (ng/mL). The MTX-treated group showed a significant increase at *p* < 0.05 in the activity of both CK and CK-MB enzymes (U/L), as well as troponin level (ng/mL) compared with the normal group (Table 1). These results indicate that MTX treatment caused toxicity in heart tissues. We then examined the protective effect of pycnogenol administration on serum heart function before MTX treatment. As indicated in Table 1, treatment of pycnogenol at different concentrations caused a significant and dose-dependent decrease at *p* < 0.05 on the serum troponin level (ng/mL) and the activity of CK and CK-MB enzymes (U/L) compared with the MTX-treated group. These results affirm the protective effect of pycnogenol toward MTX-induced toxicity on heart tissues (Figure S1).

**Figure 2:**
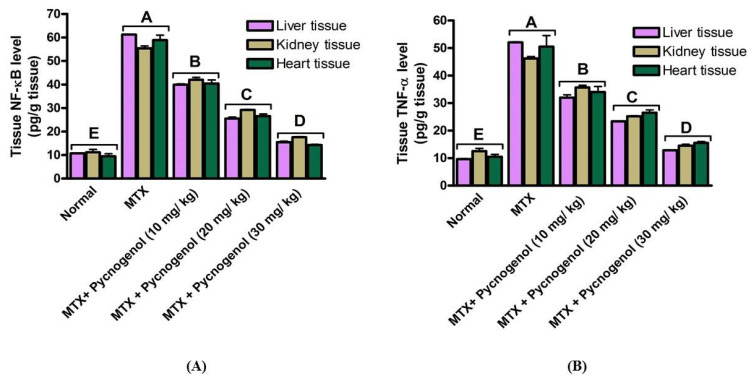
Effect of MTX and pycnogenol on (**A**) NF-κB and (**B**) TNF-α, the inflammatory biomarkers. NF-κB: nuclear factor-κB; TNF-α: tumor necrosis factor-α, *p* < 0.05. Bars represent the mean value and the standard error for means. Bars with different letters are significantly different according to DMRTs at *p* = 0.05.

**Figure 3:**
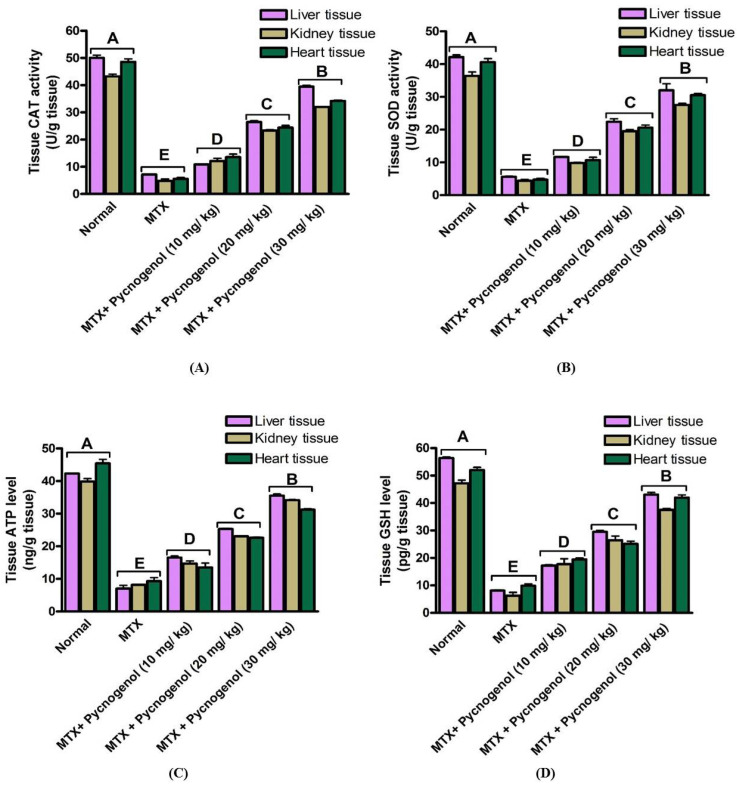
Effect of pycnogenol and/or MTX treatment on (**A**) CAT, (**B**) SOD, (**C**) ATP, and (**D**) GSH oxidative stress biomarkers (mean ± SD) in different organs. Data are mean ± SD of six rats/group. CAT: catalase, SOD: superoxide dismutase, ATP: adenosine triphosphate, GSH: reduced glutathione. *p*-value < 0.05 is significant. Bars represent the mean value and the standard error for means. Bars with different letters are significantly different according to DMRTs at *p* = 0.05.

(b) Effect of MTX and pycnogenol on tissue biomarkers:

i—Assessment of tissue inflammatory biomarkers: 

In order to examine the levels of proinflammatory cytokines, the content of NF-κB and TNF-α in liver, kidney, and heart tissue homogenate was assessed. As shown in Figure 2, the MTX-treated group demonstrated a significant increase at *p* < 0.05 in the levels of NF-κB and TNF-α (pg/g tissue) in the different investigated tissues compared with the normal group. These results indicate that MTX treatment caused inflammatory responses in the different tissues (liver, kidney, and heart). To investigate the possible anti-inflammatory effect of pycnogenol, we evaluated the level of these proinflammatory cytokines (NF-κB and TNF-α) (pg/g tissue) in the liver, kidney, and heart tissues after administration of different concentrations of pycnogenol (10, 20, and 30 mg/kg). The pycnogenol-treated groups showed a significant and dose-dependent decrease at *p* < 0.05 in the level of NF-κB and TNF-α in liver, kidney, and heart tissues compared with the MTX group (Figure 2). Notably, administration of 30 mg/kg was sufficient to reduce the level of proinflammatory cytokines to the normal level (MTX-untreated group). These results reveal the anti-inflammatory effect of pycnogenol and affirm its protective effect against MTX-induced toxicity in the different organ tissues (Figures S2–S4).

ii—Assessment of tissue oxidative stress biomarkers: 

To further investigate MTX effect, we evaluated the oxidative stress biomarkers in the liver, kidney, and heart tissues. Toward this aim, we examined the activity of CAT and SOD enzymes (U/g tissue), as well as the levels of GSH (pg/g tissue) and ATP (ng/g tissue) in the different tissues at *p* < 0.05. The results demonstrated that the MTX treatment significantly decreased the activity of CAT and SOD enzymes (U/g tissue) and attenuated the level of GSH (pg/g tissue) and ATP (ng/g tissue) in the tissues compared with the normal group (Figure 3). These findings indicate that MTX treatment induced oxidative stress in the different tissues, which influenced the mitochondria and led to a decrease in the ATP level (ng/g tissue). Furthermore, the observed reduced activity of CAT and SOD enzymes (U/g tissue) and GSH level (pg/g tissue) affirm the effect of MTX on the enzymatic and non-enzymatic antioxidant defense system of the different tissues. In contrast, the pycnogenol-treated group at different concentrations demonstrated a dose-dependent increase at *p* < 0.05 in the activity of CAT and SOD enzymes (U/g tissue) and enhanced the levels of ATP (ng/g tissue) and GSH (pg/g tissue) in the different tissues compared with the MTX group (Figure 3). Again, administration of 30 mg/kg dose was sufficient to return the oxidative stress biomarkers in the different tissues to the normal (untreated) levels. These results confirm the protective effect of pycnogenol on the MTX-induced oxidative stress in different tissues (Figures S2–S4). 

**Figure 4:**
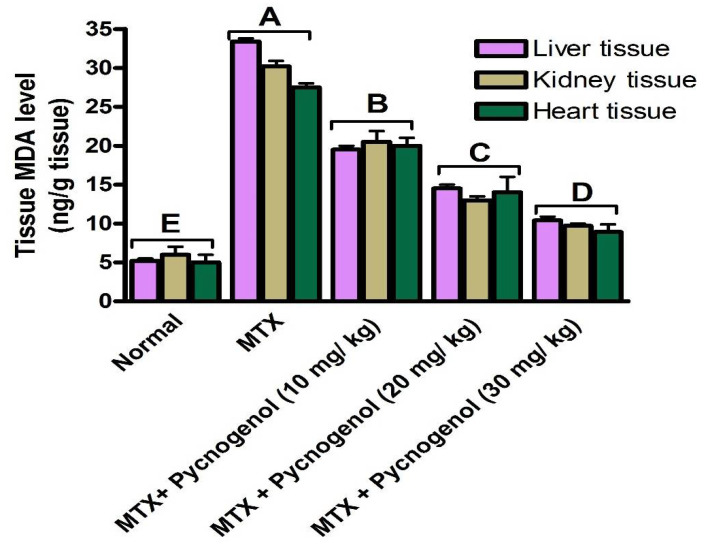
Effect of pycnogenol and/or MTX treatment on the level of MDA biomarker (mean ± SD) in the different tissues. Data are mean ± SD of six rats/group. MDA: malondialdehyde. *p* value < 0.05 is significant. Bars represent the mean value and the standard error for means. Bars with different letters are significantly different according to DMRTs at *p* = 0.05.

To affirm our findings of the effect of pycnogenol on the MTX-induced oxidative stress, we assessed the lipid peroxidation marker, MDA level (ng/g tissue), in the liver, kidney, and heart tissues. As illustrated in Figure 4, MTX treatment significantly increased (*p* < 0.05) the level of MDA (ng/g tissue) compared with the normal group. These results, in agreement with previous reports, confirm the effect of MTX on the antioxidant system, which led to an increase in the lipid peroxidation of different tissues. To further explore the antioxidant activity of pycnogenol, we evaluated the MDA biomarker in the liver, kidney, and heart tissues of the pycnogenol-treated groups. Consistent with our previous findings, administration of pycnogenol significantly (*p* < 0.05) and dose-dependently reduced the MDA level in the different tissues compared with the MTX group, with 30 mg/kg as the most effective dose. Taken together, the presented findings support the protective effect of pycnogenol against MTX-induced oxidative stress and lipid peroxidation in the different tissues (Figures S2–S4).

### 2.2. Histological Analysis

In order to confirm our biochemical findings, the effect of MTX treatment on the liver, kidney, and heart tissues, as well as the possible protective effect of pycnogenol administration, was determined using light microscopic examination of the histopathological and immunohistochemical changes. 

#### 2.2.1. Histopathologic Analysis: Examination of H&E-Stained Sections for Liver, Kidney, and Heart to Examine the Architecture of the Cells

**Figure 5:**
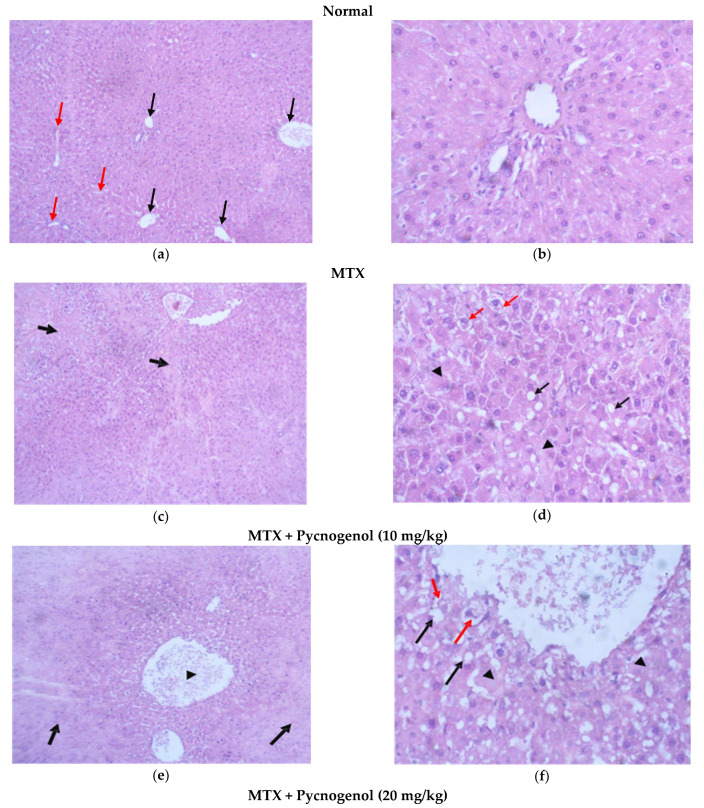

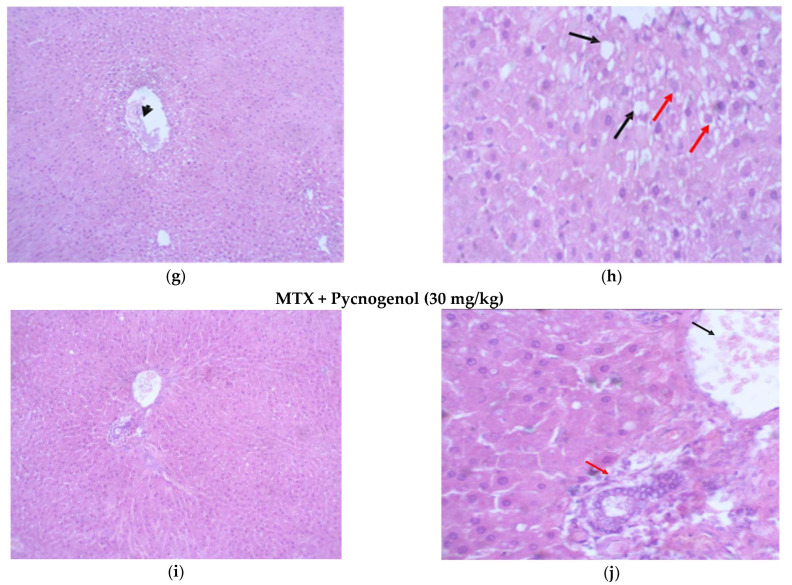
Photomicrograph of H&E-stained liver sections. (**a**) Normal liver architecture, with central vein (black arrow) radiating from plates of hepatocytes; separated by sinusoids (Red arrows) (H&E, 10x). (**b**) Higher magnification of hepatocytes with unremarkable pathological changes (H&E, 40x), (**c**) Multiple areas of confluent necrosis with occasional porto-central bridging necrosis (black arrows) (H&E, 10x). (**d**) Higher magnification of hepatocytes showing evidence of hepatocyte injury: fatty change (black arrows), hydropic degeneration (red arrows), and foci of lytic necrosis (arrowhead) (H&E, 40x). (**e**) Large area of confluent necrosis (black arrows) around congested central vein (arrowhead) (H&E, 10x). (**f**) Higher magnification of hepatocytes showing evidence of hepatocyte injury: fatty change (black arrows), hydropic degeneration (Red arrows), and foci of lytic necrosis (arrowhead) (H&E, 40x). (**g**) Normal liver architecture with mildly congested central (arrowhead) vein with no evidence of necrosis (H&E, 10x). (**h**) Higher magnification of hepatocytes shows the presence of mild fatty changes (black arrows) and hydropic degeneration (red arrows) (H&E, 40x), (**i**) normal liver architecture (H&E, 10x). (**j**) No evidence of hepatocyte injury. Central vein (black arrow) is shown, as well as a portal tract (red arrow) (H&E, 40x).

**Figure 6:**
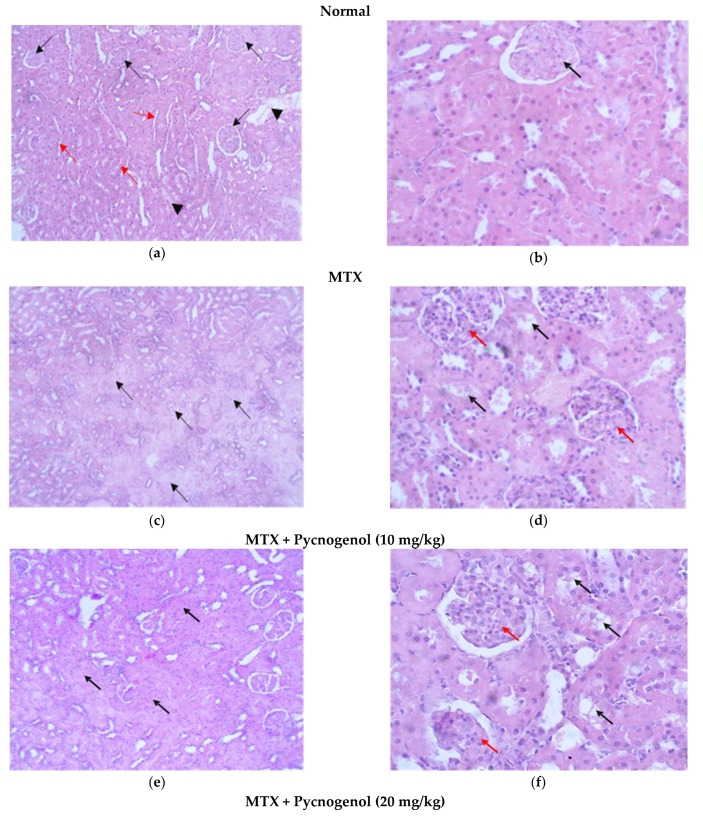

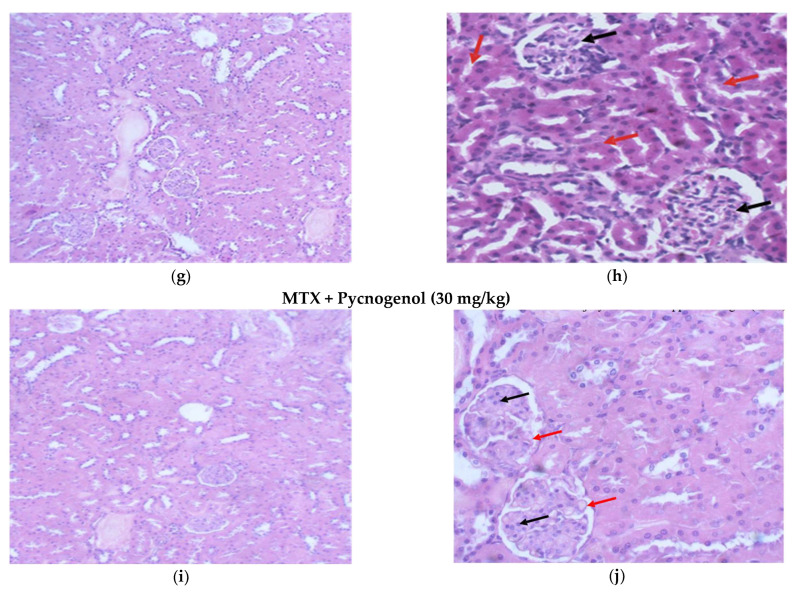
Photomicrograph of H&E-stained kidney sections. (**a**) Uniform kidney tissue, with regular glomeruli (black arrows), tubules (Red arrow), and densely packed interstitium with blood vessels (Arrow heads) (H&E, 10x). (**b**) Higher magnification of the previous figure showing uniform glomerular tuft, with normal wall thickness and patent capillary lumens (black arrow). Surrounding tubules reveal no signs of injury. (H&E, 40x). (**c**) Multiple foci representing more than 50% of tubules show evidence of tubular injury (black arrows) (H&E, 10x). (**d**) Tubules demonstrate evidence of acute tubular injury: disturbed tubular outline, loss of brush border, detached lining cells into lumen, cytoplasmic vacuolization, and membrane blebbing (black arrows). Glomeruli appear large due to mesangial expansion with mild thickening of the capillary wall (red arrows) (H&E, 40x). (**e**) Multiple foci representing less than 50% of tubules show evidence of tubular injury (black arrows) (H&E, 10x). (**f**) Some tubules demonstrate evidence of acute tubular injury (black arrows). Glomeruli show mesangial expansion with mild thickening of the capillary wall (red arrows) (H&E, 40x). (**g**) No evidence of tubular injury. Glomeruli appear enlarged (H&E, 10x). (**h**) Glomeruli (black arrows) showing mesangial hypercellularity. The tubules (red arrows) are tightly packed, with uniform epithelial lining and uniform cytoplasm (H&E, 40x). (**i**) No evidence of tubular injury. Glomeruli appear enlarged (H&E, 10x). (**j**) Glomeruli show mesangial expansion (arrow heads) with mild thickening of the capillary wall (red arrows). The tubules are tightly packed, with uniform epithelial lining and uniform cytoplasm (H&E, 40x).

**Figure 7:**
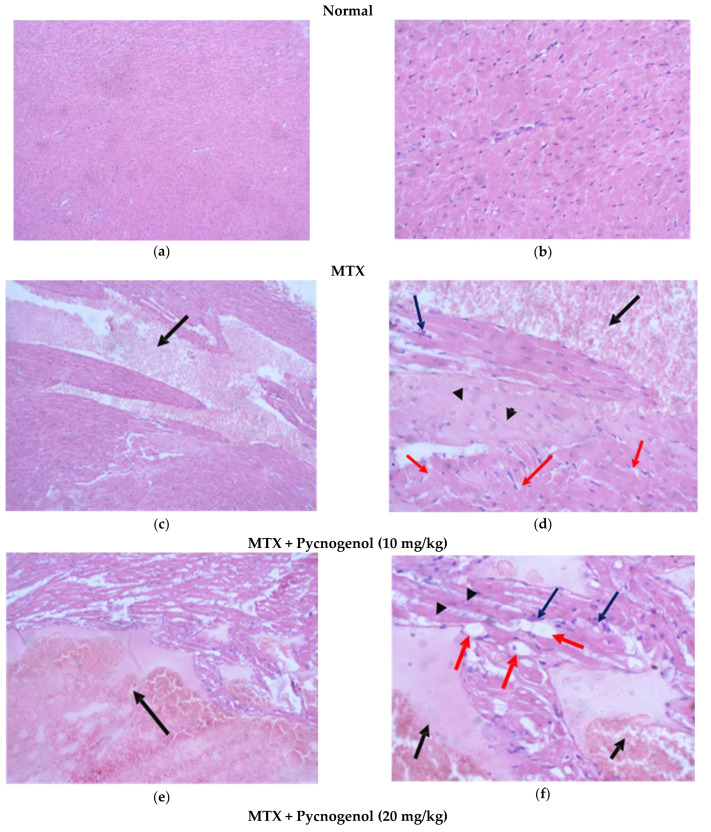

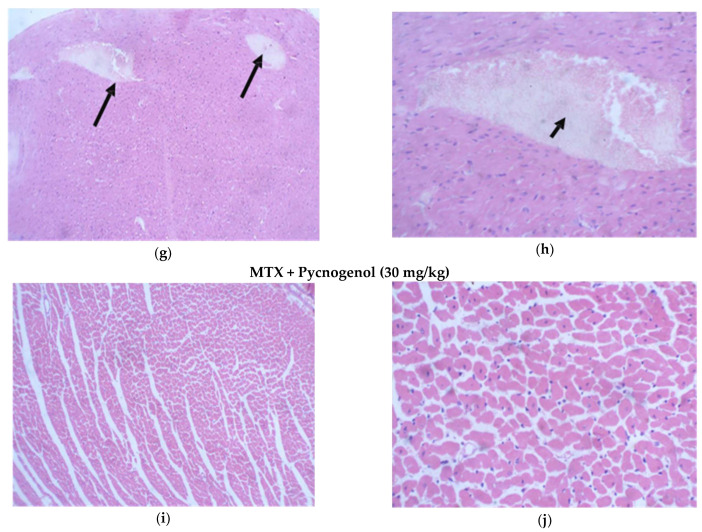
Photomicrograph of H&E-stained heart sections. (**a**) Normal heart muscle tissue (H&E, 10x). (**b**) Higher magnification of the previous figure showing uniform muscle bundles and cells (H&E, 40x). (**c**) An area of hemorrhagic myocyte necrosis representing about 20% (black arrow) muscle tissue (H&E, 10x). (**d**) Higher magnification of the previous figure showing area of hemorrhagic necrosis (black arrow), myocyte vacuolization (red arrows), and disappearance of nuclei in some myocytes (arrow heads). There is mild lymphocytic infiltrate (blue arrows) (H&E, 40x). (**e**) An area of hemorrhagic myocyte necrosis (black arrow) representing about 20% of muscle tissue (H&E, 10x). (**f**) Higher magnification of the previous figure showing an area of hemorrhagic necrosis (black arrows), myocyte vacuolization (red arrows), and disappearance of nuclei in some myocytes. There is mild lymphocytic infiltrate (blue arrows) (H&E, 40x). (**g**) Marked reduction in necrotic area, where scattered foci were evident (black arrows), representing less than 10% of muscle tissue (H&E, 10x). (**h**) Higher magnification of the previous figure showing the small area of hemorrhagic necrosis (black arrow), with no other signs of myocyte injury (H&E, 40x). (**i**) Restoration of normal heart muscle appearance (H&E, 10x). (**j**) Higher magnification of heart tissues showing no evidence of tissue injury (H&E, 40x).

i—Examination of H&E-stained sections of the liver:

Stained H&E liver sections of the normal group showed normal hepatic cell architecture, each with a well-defined cytoplasm, prominent nucleus, nucleolus, and a well-defined central vein; the hepatocyte stands radiated from it separated by sinusoids with unremarkable pathological changes (Figure 5a,b). Stained H&E liver sections of the group treated with MTX showed obvious histological damage, and the architecture of the tissues was impaired, as there were multiple areas of confluent necrosis with occasional porto-central bridging necrosis in the stained sections. Moreover, hepatocytes showed evidence of injury as fatty changes, hydropic degenerations, and foci of lytic necrosis (Figure 5c,d). In groups treated with pycnogenol prior to MTX treatment with gradient concentrations of pycnogenol, the stained H&E liver sections revealed the hepatoprotective effect of pycnogenol, as there was an improvement in liver architecture, with no sign of hepatocyte injury, a decrease in fatty changes, hydropic degeneration in the hepatocytes, and no evidence of necrosis in a dose-dependent manner (Figure 5e–j). 

ii—Examination of H&E-stained sections of the kidney:

Stained H&E kidney sections of the normal group showed renal corpuscles with regular glomeruli and an intact Bowman’s capsule with proximal and distal convoluted tubules with normal wall thickness and patent capillary lumens; surrounding tubules reveal no signs of injury (Figure 6a,b). Stained H&E kidney sections of the group treated with MTX showed obvious histological damage, the architecture of the tissues was impaired, and tubular injuries appeared as multiple foci in the tubules. In addition, the tubular outlines were disturbed, the brush borders were lost, the linings of cells was detached from the lumen, and cytoplasmic vacuolization and blebbing of the cell’s membrane occurred. Moreover, there was a mesangial expansion with mild thickening of the capillary wall, leading to enlargement of glomeruli (Figure 6c,d). Stained H&E kidney sections in groups treated with pycnogenol before MTX treatment with gradient concentrations of pycnogenol proved the renal protective effect of pycnogenol. The multiple foci percentage of pycnogenol decreased in a dose-dependent manner, indicating the decrease in the tubular injury. The tubules were tightly packed, with uniform epithelial lining and uniform cytoplasm; glomeruli appeared enlarged and showed mesangial expansion with mild thickening of the capillary wall (Figure 6e–j).

iii—Examination of H&E-stained sections of the heart:

Stained H&E heart sections of the normal group showed regular cell distribution and a normal myocardium architecture with uniform muscle bundles; cells had central nuclei (Figure 7a,b). Administration of MTX caused impairment in the architecture of cardiac tissues, with the appearance of a small percentage of hemorrhagic myocyte necrosis, myocyte vacuolization, and disappearance of nuclei in some myocytes. Moreover, lymphocytes were infiltrated in some areas of cardiac tissues (Figure 7c,d). Administration of pycnogenol in different concentrations affirmed the cardioprotective effect in a dose-dependent manner. Treatment with pycnogenol caused a decrease in the necrotic areas with no signs of myocyte injury and restoration of normal cardiac appearance (Figure 7e–j).

To further evaluate our histological analysis, we estimated the histological score for the tissue injuries by different scoring indices, as shown in Table 2. No injuries were found in the tissues of the normal group; on the other hand, administration of MTX resulted in histological changes in the liver, kidney, and heart tissues. In the liver tissues treated with MTX, there was obvious confluent necrosis and multiple portal-central bridging, with one focus or less per 10X objective lytic necrosis in the hepatocytes, which also caused fatty changes and the appearance of hydropic degeneration for kidney tissues treated with MTX; there was Acute tubular injury in more than 50% of tubules injury, 25–50% mesangial expansion, and sclerotizing glomerulus. MTX treatment also affected the heart tissues, as it caused mild myocyte vacuolization, myocyte necrosis, and infiltration for the mononuclear cells compared to the normal group. All these data affirm the toxicity of MTX in liver, kidney, and heart cells. Groups protected by the administration of pycnogenol with different doses (10, 20, and 30 mg/kg) showed improvement in these scores and injuries in a dose-dependent manner in the hepatocytes, kidney, and myocytes, with the high dose (30 mg/kg) being the most effective dose compared with the MTX group.

#### 2.2.2. Immunohistochemical Analysis

**Figure 8:**
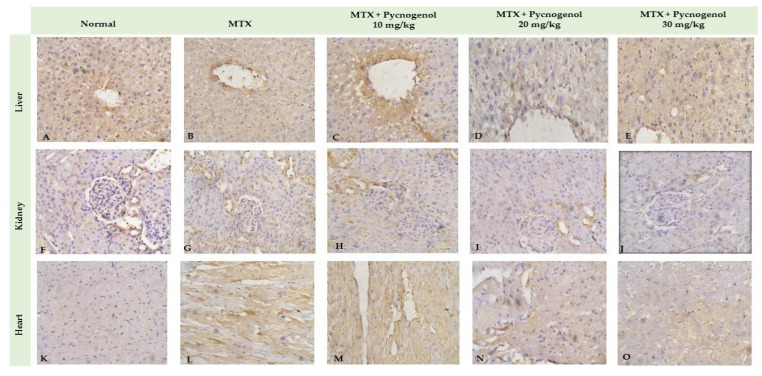
Immunohistochemical staining with IHC-caspase 3 of liver (**A**–**E**), kidney (**F**–**J**), and heart (**K**–**O**) tissue. Differences assessed by Kruskal–Wallis test at *p* = 0.05.

**Figure 9:**
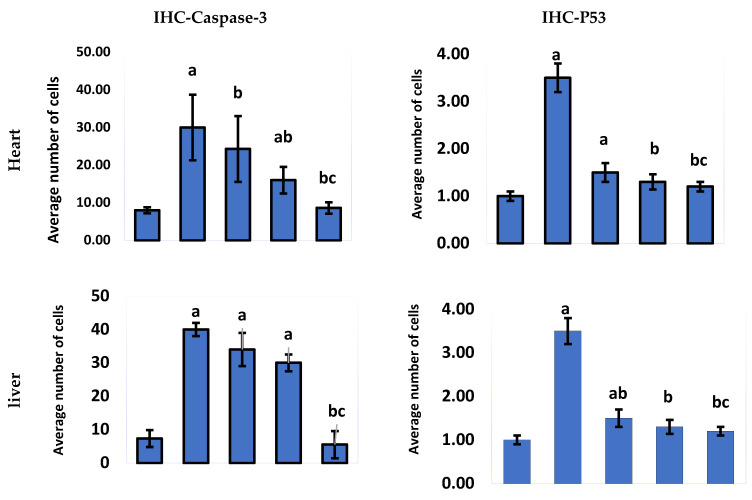

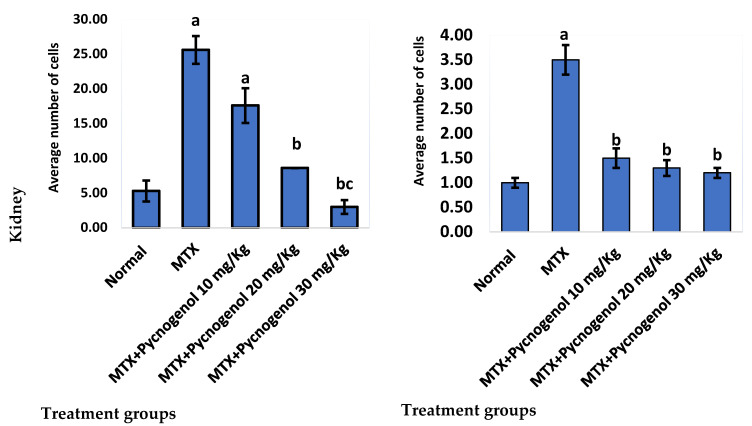
Immunohistochemical staining with IHC-caspase 3 and IHC-P53 of heart, liver, and kidney tissue. Differences assessed by Kruskal–Wallis test at *p* = 0.05. ^a^ Significantly different from normal group. ^b^ Significantly different from MTX group. ^c^ Significantly different from MTX + pycnogenol (10 mg/kg).

**Figure 10:**
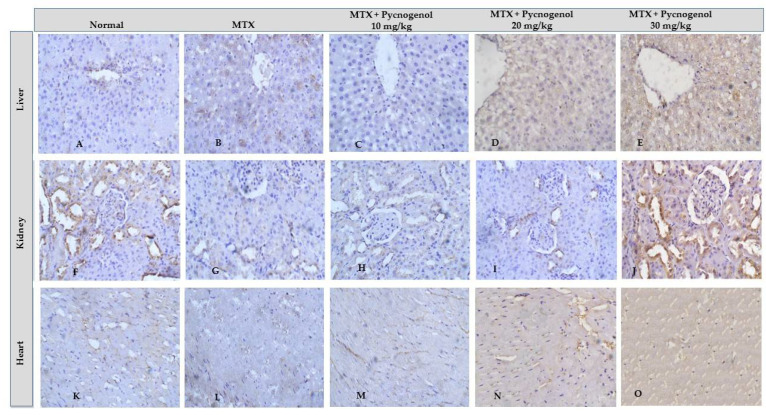
Immunohistochemical staining with IHC-P53 of heart (**A**–**E**), liver (**F**–**J**), and kidney (**K**–**O**) tissue. Differences assessed by Kruskal–Wallis test at *p* = 0.05.

In the current study, we tested pycnogenol against MTX-treated animal tissues to detect the pycnogenol protective effects. A positive reaction to IHC-caspase 3 was detected in the heart, liver tissues, and renal tubules. Adding pycnogenol to MTX-treated tissues resulted in a decrease in the expression of caspase 3 in the heart, liver, and kidney tissues in a dose-dependent manner compared to the MTX-treated group (Figure 8 and Figure 9). The current study shows that the normal group has mild (heart, liver, and kidney) IHC-P53 expression. The control group demonstrated moderate IHC-p53 expression in the heart, liver, and kidney tissues. Pycnogenol-treated groups with different pycnogenol doses (10, 20, and 30 mg/kg) showed a significant decrease in IHC-P53 expression level in heart, liver, and renal tissues in comparison with the MTX-treated group; results obtained from the higher dose (30 mg/kg) were the best (Figure 9 and Figure 10).

## 3. Discussion

The application of anticancer chemotherapy drugs has remained the mainstay treatment of malignant disease. Among different anticancer drugs, MTX is a folate antagonist that is extensively used to treat rheumatoid arthritis and cancer [39]. MTX is commonly used as an antimetabolite in cancer chemotherapy and causes an indirect decrease in DNA synthesis [40]. MTX predominantly suppresses cancer cell proliferation by inhibiting dihydrofolate reductase, a key enzyme in cell survival and proliferation [41]. The cytotoxicity of MTX was correlated with ROS production, resulting in oxidative stress and apoptosis [40]. Continuous MTX administration showed cytotoxic effects that were not limited to cancer cells but extended to affect vital organs, including the heart [42]. MTX therapy induces oxidative stress and inflammatory pathways in non-target cells and subsequent tissue damage [43,44]. 

Based on these findings, we intended to examine the molecular and cellular effects of MTX on the cellular dysfunction of the liver, kidney, and heart tissues. Toward this aim, rats were treated with MTX and then subjected to a variety of biochemical, histological, and immunohistochemical examinations. Treatment with MTX resulted in an increase in the serum ALT and AST enzyme activities (vital biomarkers of liver diseases) [45]. The observed effect of MTX on ALT and AST enzymes could be attributed to MTX-induced oxidative stress and lipid peroxidation, which led to damage to the liver and disruption of the hepatocyte bilipid layer [40,46]. The liver and kidney the most severely affected organs by MTX-induced cell damage [47]. Our results also showed an increase in the serum urea and creatinine levels in the MTX-treated group. These results may be due to the renal dysfunction caused by the precipitation of MTX and its metabolites inside the renal tubules. In addition, the tissue damage caused by ROS production and oxidative stress can lead to changes in cellular function and tissue damage [48,49]. MTX-induced cytotoxicity influenced the heart cells and resulted in the release of cardiac biomarkers such as troponin, CK, and CK-MB into the serum [15]. Consistent with previous reports, our results show that MTX treatment significantly increased the release of troponin, CK, and CK-MB into the serum, which led to cytotoxicity in the heart cells [15]. Furthermore, we examined the cytotoxic effects of MTX on inflammatory biomarkers in the liver, kidney, and heart tissue homogenates. The results indicate that hepatic, renal, and cardiac NF-κB and TNF-α levels were significantly increased in the MTX-treated group. These results suggest that the MTX-mediated inflammatory pathway is mostly dependent on NF-κB transcription and TNF-α elevation [50]. Next, we investigated the oxidative stress indicators in liver, kidney, and heart tissue homogenates. In our investigations, we observed a significant drop in the activity of the CAT and SOD enzymes, as well as a decrease in the levels of GSH and ATP in the MTX-treated groups. These results are in agreement with previous studies that reported that MTX treatment led to reduced GSH, SOD, and CAT activity, as well as elevated MDA [50,51,52]. Notably, the magnitude of GSH detected (pg/g tissue) in our experiments is lower than previously reported studies values. This could be attributed to several conditions, including age of experimental animals, environmental conditions, duration of experiments, and the amount of enzymes produced. 

Several stimuli, including hormones, cytokines, growth factors, and immunological responses, can influence the apoptosis process. The presence of cells expressing active caspase 3 is regarded as a marker of apoptotic activation [53,54]. Furthermore, it has been demonstrated that the p53 proteins play a critical regulatory role in apoptosis [40,53]. In our investigations, the immunohistochemical analysis indicated that the MTX-treated group exhibited a significant upregulation of p53 and caspase 3. The anticancer mechanism of MTX is mainly based on the initiation of p53-dependent apoptosis [55]. Accumulation of p53 protein is probably a predictor of response to MTX-induced inflammation and oxidative stress in provoking apoptosis. Furthermore, histological analysis affirmed the cytotoxic effect of MTX on hepatic, renal, and cardiac tissues. The architecture of the cells was disturbed, and necrotic markers appeared in the histological analysis of the MTX-treated group. MTX induces oxidative stress and lipid peroxidation, which cause damage to the cell membrane and membrane integrity [46].

Several studies have reported the extensive use of natural compounds or their derivatives as sources for the development of anticancer drugs [56]. Plant polyphenols serve as natural antioxidants through a variety of processes, including free-radical scavenging, metal chelation, and protein binding [30]. Among natural polyphenols, pycnogenol is an extract of a generic French pine bark (*Pinus pinaster Aiton*) that contains polymeric flavonoids (70%) and monomeric flavonoids (30%) [31]. The high concentration of flavonoids in pycnogenol is could be attributed to the wide range of antioxidant effects against both ROS and nitrogen species [43]. Moreover, pycnogenol exhibits anti-inflammatory [57] and anticancer activity [58,59]. Given the promising activity of pycnogenol, with the current study, we aimed to investigate the possible protective effect of pycnogenol against MTX-induced toxicity. Toward this aim, pycnogenol was applied at different doses (10, 20, and 30 mg/kg) before MTX was administrated, and the effect of pycnogenol was examined by biochemical, histopathological, and immunochemical analyses (Figure 2, Figure 3, Figure 4, Figure 5, Figure 6, Figure 7, Figure 8, Figure 9 and Figure 10). 

Pre-treatment with pycnogenol resulted in significant protection of liver cells against different injurious agents, as indicated by the ability of pycnogenol to restore the serum biomarkers levels of AST and ALT toward the normal range, suggesting the protective role of pycnogenol in liver cells [30]. The protective effect of pycnogenol on the kidney was also revealed in blood urea and creatinine as biomarkers of renal salvage [60]. The protective effect of pycnogenol on the heart, CK, and CK-MB biomarkers, has been discussed in several studies, which reported the improvement of physiological and electrical heart functions with pycnogenol treatment [48,61,62]. Troponin levels were also reduced upon synergetic pycnogenol treatment with cisplatin [63]. In agreement with these results, in our study, administration of pycnogenol to the control group (MTX-treated) led to a decrease in heart biomarkers, CK and CK-MB, and reduced troponin levels toward normal levels in the healthy group.

Pycnogenol also acts as an antioxidant and protective agent for several disorders [43,64,65]. In our investigations, treatment with pycnogenol significantly increased the level of GSH, SOD, and CAT enzymes, with a significant decrease in MDA levels in the MTX-treated group. Ozoner et al. reported that the oxidative stress and the oxidative-stress-associated damage in cardiac tissues could be attributed to the GSH depletion and that pycnogenol treatment proved to reverse the effect of GSH depletion on cardiac tissue [66]. Moreover, the oxidative-stress-related enzymes, including SOD, GSH, and CAT, in kidney tissue suffering from oxidative stress were found to enhance upon pycnogenol treatment, whereas MDA decreased simultaneously [67]. Our results suggest a protective effect of pycnogenol against the MTX-induced oxidative effect on the non-target heart, liver, and kidney tissue by restoring the level of the oxidative stress markers in the serum and tissues to almost normal levels.

Pycnogenol showed a wide range of anti-inflammatory effects, thus protecting against proinflammatory disorders [68]. The mode of action of pycnogenol was proposed to be mediated by the downregulation of the activated inflammatory pathway, including NF-κB and TNF-α, as well as the elevation of ATP [58,69]. Consistent with this anti-inflammatory mechanism, our study showed that pycnogenol treatment significantly decreased the level of NF-κB and TNF-α and increased ATP levels in the MTX-treated group. In previous studies, the effective dose of pycnogenol used as an antioxidant and anti-inflammatory agent ranged from 50 mg/kg [43,67] to 200 mg/kg [60]. The current study showed similar results, although at a lower effective dose of 30 mg/kg. Pycnogenol reduced NF-κB and other proinflammatory cytokines in brain [70], lung [71], joint [68], and kidney tissues [72]. In the current study, we extended previous studies by examining NF-κB, TNF-α, and ATP levels in liver, kidney, and heart tissues. Our results are in line with those reported in previous studies in that pycnogenol treatment reduced the proinflammatory mediators, including NF-κB and TNF-α, and elevated protective ATP, thus reversing the adverse effect of MTX [67]. Accordingly, these results indicate that pycnogenol treatment has a protective role in vital organs by reducing the inflammatory pathway induced by MTX therapy. 

**Figure 11:**
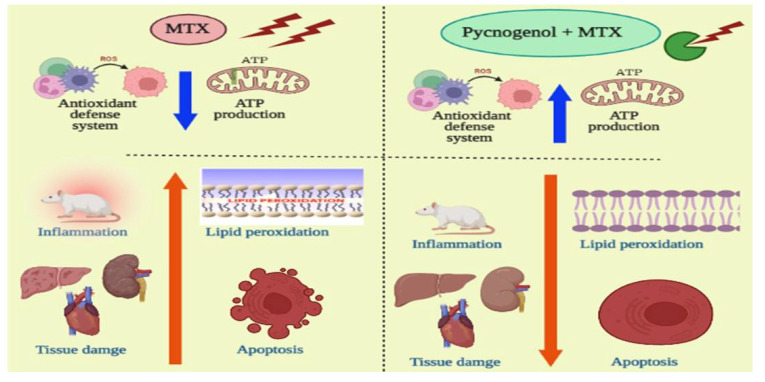
Illustration of the protective effect of pycnogenol on MTX-induced toxicity in different organs.

To further confirm our biochemical analysis, we investigated the protective effect of pycnogenol on different organs by performing detailed histopathological and immunochemical analyses. Our immunohistochemical analysis revealed that pycnogenol treatment had an ameliorative effect on the expression of p53 and caspase 3 proteins. These results could be attributed to the protective and antioxidant effect of pycnogenol, which decreases ROS and enhances the antioxidant defense system, suppressing the apoptotic pathway. Moreover, our histological studies revealed that the architecture of the hepatic, renal, and cardiac tissues was improved with pycnogenol treatment, suggesting the potent antioxidant and anti-inflammatory effects of pycnogenol Figure 11. 

## 4. Materials and Methods

### 4.1. Animals and Grouping:

All animal experiments were approved by the Institutional Animal Ethics Committee of Suez Canal University, Faculty of Pharmacy. Thirty male Wistar rats with a mean weight of 150 g were housed in an animal house with temperature (22 °C) and lighting control (12 h light–12 h dark cycle). An adaptation period of 1 week was allowed before the initiation of the experimental protocol. The rats were randomly distributed equally into four groups (*n* = 6):

Group I rats were injected daily with a saline vehicle [1:9 DMSO/saline mixture, 0.2 mL/rat, i.p.] and were considered as a vehicle (control/normal) group.

Group II rats were injected with 0.2 mL of the vehicle (1:9 DMSO/saline mixture) for 14 days; then, the rats were given a single dose of MTX (10 mg/kg, i.p.). This group served as the MTX control group.

Group III, IV, and V rats were orally treated for 14 successive days with pycnogenol (10, 20, and 30 mg/kg, 1:9 mixture of DMSO and saline, 0.2 mL/rat, P.O., oral gavage) [69,73]. Then, the rats were administered a single dose of MTX (10 mg/kg, i.p.) [74].

### 4.2. Chemicals and Reagents

Pycnogenol extract was purchased from Clearsynth Ltd. (Clearsynth Ltd., Mississauga, Toronto, Canada). DMSO, saline, and MTX were purchased from Sigma-Aldrich (Germany).

### 4.3. Induction of Toxicity by MTX

After adaptation for 6–7 days, animals were treated with vehicle or pycnogenol for 14 days (10, 20, and 30 mg/kg, P.O, oral gavage). Then, they were intraperitoneally injected with a single dose of MTX (10 mg/kg, i.p.). All rats had access to regular standard rat chow during the treatment period.

### 4.4. Collection of Blood and Tissue Samples

Rats were sacrificed 72 h after MTX injections under ketamine anesthesia. Then, blood samples were collected in a dry Eppendorf tube and centrifuged for 10 min at 1200 g after clotting at ambient temperature to afford the sera. Serum samples were then split into different tubes and preserved at −20 °C for later evaluation of liver enzyme activities (AST & ALT), creatinine, urea, and troponin levels, as well as activities of CK and CK-MB. After performing a laparotomy, the left ventricle of the heart, right lobe of the liver, and right kidney were kept in paraformaldehyde solution (10%) and used for histological investigations. Then, the previously selected tissues were removed and thoroughly cleaned with ice-cold normal saline before being kept at −80 °C. Homogenization of frozen tissues samples in phosphate-buffered saline (PBS) (10% *w/v*, 50 mmol/L, pH = 7.4) was performed utilizing a Teflon homogenizer (Glas Col homogenizer system, Vernon Hills, IL, USA). Later, this homogenate was utilized to assay for ATP tissue level; oxidative stress indicators, such as malonaldehyde (MDA) level, enzymatic antioxidant activities (CAT & SOD), and non-enzymatic antioxidant (GSH) levels; as well as inflammatory markers, such as TNF-α and NF-κB.

### 4.5. Biochemical Analysis

(a) Serum biomarkers:

i—Assessment of liver function:

To evaluate the functions of the liver, we estimated liver enzyme activities in the serum, and the activities of liver enzymes AST and ALT were calculated using marketed kits (Biocon Diagnostic, Vöhl, Germany, CA 92807 1-800-222-9880). The manufacturer’s protocol was used as a guide to use these kits.

ii—Assessment of kidney function:

Serum creatinine and urea levels assist in the evaluation of renal function. Creatinine levels were determined utilizing a diagnostic kit’s alkaline picric acid technique (Diamond Diagnostics Ltd., Cairo, Egypt, CA 94545). Serum urea was determined using a diagnostic kit reaction with salicylate-hypochlorite reagent in the presence of the catalyst nitroprusside to produce a green indophenol (Diamond Diagnostics Ltd., Cairo, Egypt). A spectrophotometer was used to determine the optical densities of the reactions (UV1601-PC, Shimadzu, Japan).

iii—Assessment of heart function:

To estimate the damage caused by hepatic tissue injury, serum creatine phosphokinase (CK), creatine kinase-MB isoenzyme (CK-MB), and troponin levels were measured using enzymatic methods with an ultraviolet-visible spectrophotometer (UV-1601PC, Shimadzu, Japan) and commercial kits purchased from Stanbio (MBS727624, TX, USA).

(b) Tissue biomarkers:

i—Assessment of tissue inflammatory biomarkers: 

To evaluate the expression of proinflammatory cytokines, the content of NF-κ and TNF-α in cardiac, hepatic, and renal homogenate was assessed using enzyme-linked immunosorbent assay (ELISA) kits. TNF-α was obtained from RayBiotech Inc. (3607 Parkway Lane, Suite 200 Norcross, Peachtree Corners, GA, USA), and NF-κB was obtained from EIAab™ (East Lake Hi-Tech Development Zone, Wuhan, China); both were used according to the recommendations of the manufacturer.

ii—Assessment of tissue oxidative stress biomarkers:

To explore the oxidative stress level, a part of cardiac, hepatic, and renal homogenate was used for determination of MDA levels using a colorimetric method depending on the reaction with thiobarbituric acid, as described previously [75,76], whereas GSH level was measured using a colorimetric method depending on the reduction of 5,5 dithiobis (2- nitrobenzoic acid) (DTNB) with GSH, as described previously [77]. The methods were a using UV dithiobis assay kits for ATP, SOD, and CAT in accordance with the instructions of the manufacturer (MBS036924, Biodiagnostic, Egypt). 

### 4.6. Histological Analysis:

(a) Histopathologic analysis:

Histopathologic examinations were performed on the heart, liver, and kidneys. After fixation overnight in 10% neutral buffered formalin, the tissues were dehydrated in a series of increasing alcohol concentrations and embedded in paraffin. Subsequently, four-millimeter-thick slices were cut and administered for normal hematoxylin and eosin staining (H&E staining). The stained sections were then examined under a light microscope [78].

i—Histopathological study of liver tissue sections: 

Hepatic lesions were identified according to the following tables (Table 3, Table 4 and Table 5) [79]. 

ii—Histopathological study of kidney tissue sections: 

Renal glomerular and tubular damage were scored by a semi-quantitative method according to [80], wherein acute tubular injury changes were used to score the degree of renal tubular damage, including disturbed tubular outline, loss of brush border, detached lining cells into lumen, cytoplasmic vacuolization, and membrane blebbing [81]. 

iii—Histopathological study of heart tissue sections

Heart sections were microscopically evaluated for degenerative myocardial changes of. The severity of observed microscopic lesions was graded based on the degree and extent of tissue damage using a four-point scale [82]. The cardiac tissue was assessed according to the presence of the following lesions: myocyte vacuolization, myocyte necrosis, mononuclear cell infiltration, and fibrosis. 

(b) Immunohistochemical analysis

Immunohistochemical staining was carried out for P53 and caspase-3 in cardiac, hepatic, and renal tissues using a commercially available labelled streptavidin–biotin–peroxidase complex method. To evaluate immunohistochemistry for P53 and caspase-3, slides were scanned at x40 magnification, and ten cellular zones were identified (referred to as hot spots) and photographed at x400 magnification for additional assessment. The slides were examined using an Olympus CX31 light microscope, and pictures were taken using a computer-driven digital camera (Olympus E-620) [83,84]. 

### 4.7. Statistical Analysis

The sample size was identified using G power program (n = 6). Data were analyzed with ANOVA analysis utilizing SPSS.21 software (IBM Inc. Chicago, IL, USA). Data were gathered and revised, and then statistical analysis was applied using one-way ANOVA. Data were described as mean ± standard deviation (SD). The data significance was defined by *p*-value (*p* > 0.05, non-significant; *p* < 0.05, significant). Statistical significance between different groups was defined by applying one-way ANOVA, followed by Duncan’s multiple range (DMRTs) *post hoc* test. Furthermore, the discrete data were analyzed using the Kruskal-Wallis test, followed by the Mann-Whitney U test. 

## 5. Conclusions

Although MTX is a potent anticancer and anti-inflammatory medication, its untoward effects on non-target cells may limit the use of MTX and increase the rate of MTX discontinuation. Therefore, several studies were conducted to identify a suitable adjunctive agent to counteract the untoward effects of MTX. Pycnogenol has been proven to induce antioxidant and anti-inflammatory effects, as well as antitumor influence. Pycnogenol was concluded to act as an antioxidant agent in different situations, including heat stress. The improvement of the cardiac biomarkers is evidence of the protective effect of pycnogenol on the integrity of heart cells, and the reversal of the deleterious effect of MTX on heart tissue. To the best of our knowledge, this is the first study to investigate the potential protective features of pycnogenol in order to alleviate untoward MTX-mediated effects on non-target cells. Pycnogenol plays a salient role in preserving cell architecture and physiological functions, in addition to preventing adverse reactions related to MTX treatment. The antitumor effect is an additive feature. Pycnogenol was proven to have significant protective value and may decrease MTX withdrawal to a considerable extent.

## Figures and Tables

**Table 1:** Effect of MTX and pycnogenol treatment on serum biomarkers.

Parameter	Normal Group	MTX Group	MTX + Pycnogenol (10 mg/kg) Group	MTX + Pycnogenol (20 mg/kg) Group	MTX + Pycnogenol (30 mg/kg) Group
ALT (U/L)	35.33 ± 2.5 ^E^	104.00 ± 4.0 ^A^	84.00 ± 2.0 ^B^	61.00 ± 1.0 ^C^	46.00 ± 1.0 ^D^
AST (U/L)	41.03 ± 0.2 ^E^	136.93 ± 3.6 ^A^	101.00 ± 3.0 ^B^	78.00 ± 2.0 ^C^	55.67 ± 2.5 ^D^
Urea (mg/dL)	36.00 ± 2.0 ^E^	64.00 ± 1.0 ^A^	51.00 ± 1.0 ^B^	43.00 ± 1.0 ^C^	39.00 ± 1.0 ^D^
Creatinine (mg/dL)	0.50 ± 0.1 ^B^	1.87 ± 0.4 ^A^	0.90 ± 0.1 ^B^	0.70 ± 0.1 ^B^	0.60 ± 0.1 ^B^
Troponin (ng/mL)	0.94 ± 0.1 ^D^	4.57 ± 0.4 ^A^	2.08 ± 0.1 ^B^	1.72 ± 0.1 ^C^	1.07 ± 0.1 ^D^
CK (U/L)	12.27 ± 0.3 ^E^	56.10 ± 0.1 ^A^	35.00 ± 0.1 ^B^	23.07 ± 0.1 ^C^	16.47 ± 0.5 ^D^
Ck-MB (U/L)	10.50 ± 0.1 ^E^	36.50 ± 0.1 ^A^	23.70 ± 0.6 ^B^	16.23 ± 1.1 ^C^	12.07 ± 0.2 ^D^

Data are presented as mean ± SD (n = 6). ALT: alanine aminotransferase; AST: aspartate aminotransferase; CK: creatine kinase; CK-MB: creatine kinase-MB isoenzyme. *p* < 0.05. Data with different letters are significantly different according to DMRTs at *p* = 0.05.

**Table 2:** Effect of MTX and pycnogenol treatment on histopathological evaluation of liver, kidney, and heart tissue sections.

Organs and Scoring Index		Normal	MTX	MTX + Pycnogenol (10 mg/kg)	MTX + Pycnogenol (20 mg/kg)	MTX + Pycnogenol (30 mg/kg)
Liver	Hepatic activity index Grading:	0	6	6	0	0
	-Confluent necrosis	Absent (0)	Necrosis and multiple portal-central bridging (5)	Necrosis and multiple portal-central bridging (5)	Absent (0)	Absent (0)
	-Lytic necrosis, apoptosis, and focal inflammation	Absent (0)	One focus or less per 10X objective (1)	One focus or less per 10X objective (1)	Absent (0)	Absent (0)
	-Piecemeal necrosis	Absent (0)	Absent (0)	Absent (0)	Absent (0)	Absent (0)
	-Portal inflammation	Absent (0)	Absent (0)	Absent (0)	Absent (0)	Absent (0)
	Other changes	No changes	Fatty change and hydropic degeneration	Fatty change and hydropic degeneration	Mild fatty change and hydropic degeneration	No evidence of hepatocyte injury
Kidney	Tubular injury	Absent (0)	Acute tubular injury in more than 50% of tubules (3)	Acute tubular injury in 10–50% of tubules (2)	Absent (0)	Absent (0)
	Glomerular injury	Absent (0)	25–50% mesangial expansion and sclerotizing glomerulus (2)	25–50% mesangial expansion and sclerotizing glomerulus (2)	25–50% mesangial expansion and sclerotizing glomerulus (2)	25–50% mesangial expansion and sclerotizing glomerulus (2)
	Total score	0	5	4	2	2
Heart	Myocyte vacuolization	Absent (0)	Mild (2)	Moderate (3)	Absent (0)	Absent (0)
	Myocyte necrosis	Absent (0)	Mild (2)	Mild (2)	Minimal (1)	Absent (0)
	Mononuclear cells infiltration	Absent (0)	Mild (2)	Mild (2)	Absent (0)	Absent (0)
	Fibrosis	Absent (0)	Absent (0)	Absent (0)	Absent (0)	Absent (0)

**Table 3:** Scoring features for lobular architectural changes and hepatic activity index (HAI) grading: necro-inflammatory.

Lobular Architecture	Score
Normal (absence of fibrosis)	0
Fibrous expansion of some portal areas	1
Fibrous expansion of most portal areas, with portal–portal septa	2
Fibrous extension of portal spaces with portal–portal and portal–central septa, with possible nodule formation	3
Cirrhosis with predominant nodular areas in relation to the remaining lobules	4
A. Periportal or periseptal interface hepatitis (piecemeal necrosis)	Degree
Absent Mild (focal, few portal areas) Mild/moderate (focal, most portal areas) Moderate (continuous, around 60% of tracts or septa) Severe (continuous, >50% of tracts or septa)	0 1 2 3 4
B. Confluent necrosis	Degree
Absent 0 Focal confluent necrosis 1 Zone 3 necrosis in some areas Zone 3 necrosis in most areas Zone 3 necrosis + occasional portal–central (P-C) bridging Zone 3 necrosis + multiple P-C bridging Panacinar or multiacinar necrosis	0 1 2 3 4 5 6
C. Focal (spotty) lytic necrosis, apoptosis, and focal inflammation	Degree
Absent One focus or less per 10x objective Two to four foci per 10x objective Five to ten foci per 10x objective More than ten foci per 10x objective	0 1 2 3 4
D. Portal inflammation	Degree
Absence of portal lymphocytes Mild number of portal lymphocytes Moderate number of portal lymphocytes Marked number of portal lymphocytes Strongly marked number of portal lymphocytes	0 1 2 3 4

**Table 4:** Scoring features for renal tubular and glomerular damage.

**Lesion**	**Grade**
No lesion	0
Acute tubular injury in 10% of tubules	1
Acute tubular injury in 10–50% of tubules	2
Acute tubular injury in more than 50% of tubules	3
Complete atrophy and thyroidization of the tubules	4
Lesion	Grade
No sclerosis	0
1–25% mesangial expansion and sclerosing glomerulus	1
25–50% mesangial expansion and sclerotizing glomerulus	2
50–75% mesangial expansion and sclerosing glomerulus	3
75–100% mesangial expansion and sclerotizing glomerulus	4

**Table 5:** Scoring features for degenerative myocardial changes.

Lesion	Grade
No lesion	0
Lesions involved less than 10% of the heart section	1
Lesions involved 11–40% of the heart section	2
Lesions involved 41–80% of the heart section	3
Lesions involved more than 81% of the heart section	4

## Data Availability

Data is contained within the article and supplementary material.

## Supplementary Materials

The following are available online at https://www.mdpi.com/article/10.3390/ph15060674/s1, Figure S1: Regression trendline showing the relationship between increasing pycnogenol levels (10, 20, and 30 mg Kg^−1^) and various serum biomarkers. Figure S2: Regression trendline showing the relationship between increasing pycnogenol levels (10, 20, and 30 mg Kg^−1^) and various heart biomarkers. Figure S3: Regression trendline showing the relationship between increasing pycnogenol levels (10, 20, and 30 mg Kg^−1^) and various liver biomarkers. Figure S4: Regression trendline showing the relationship between increasing pycnogenol levels (10, 20, and 30 mg Kg^−1^) and various kidney biomarkers.

## Abbreviations

ALP: alkaline phosphatase, ALT: alanine aminotransferase, AST: aspartate aminotransferase, ATP: adenosine triphosphate, BCA: Bicinchoninic acid, CAT: catalase, CK: creatinine kinase, CK-MB: creatinine kinase MB, GSH: reduced glytathione, iNOS: inducible nitric oxide synthase, JNK: Jun N-terminal kinase, MAPK: mitogen-activated protein kinase, MDA: malondialdehyde, MTX: methotrexate, NF-*κ*B: nuclear factor-*κ*B, ROS: reactive oxygen species, SOD: superoxide dismutase, TNF-α: tumor necrosis factor-α.
