# Supplementary material for: Protective Effect of Pycnogenol against Methotrexate-Induced Hepatic, Renal, and Cardiac Toxicity: An In Vivo Study
Source: Pharmaceuticals (Basel). 2022 May 27;15(6):674. doi: 10.3390/ph15060674 (PMC9229807; doi:10.3390/ph15060674)
Supplement: Supplementary file 1 [file pharmaceuticals-15-00674-s001.zip › pharmaceuticals-1677979-supplementary.pdf]

Supplementary Information

Insights into the Protective Effect of Pycnogenol Against Methotrexate-Induced Toxicity: *in silico* and *in vivo* study

Faten Al-Abkal[1], Basel A. Abdel-Wahab[2], Hanaa F. Abd El-Kareem[3], Yasser M. Moustafa [1,4], Dina M. Khodeer[1,*]

Supplement for regression analysis

1- Biochemical analysis:

a) Serum biomarkers:

i. Liver function: activities of ALT, AST enzymes

ii. Kidney function: level of urea and creatinine

iii. Heart function: level of troponin and activities of CK and CK-MB enzymes

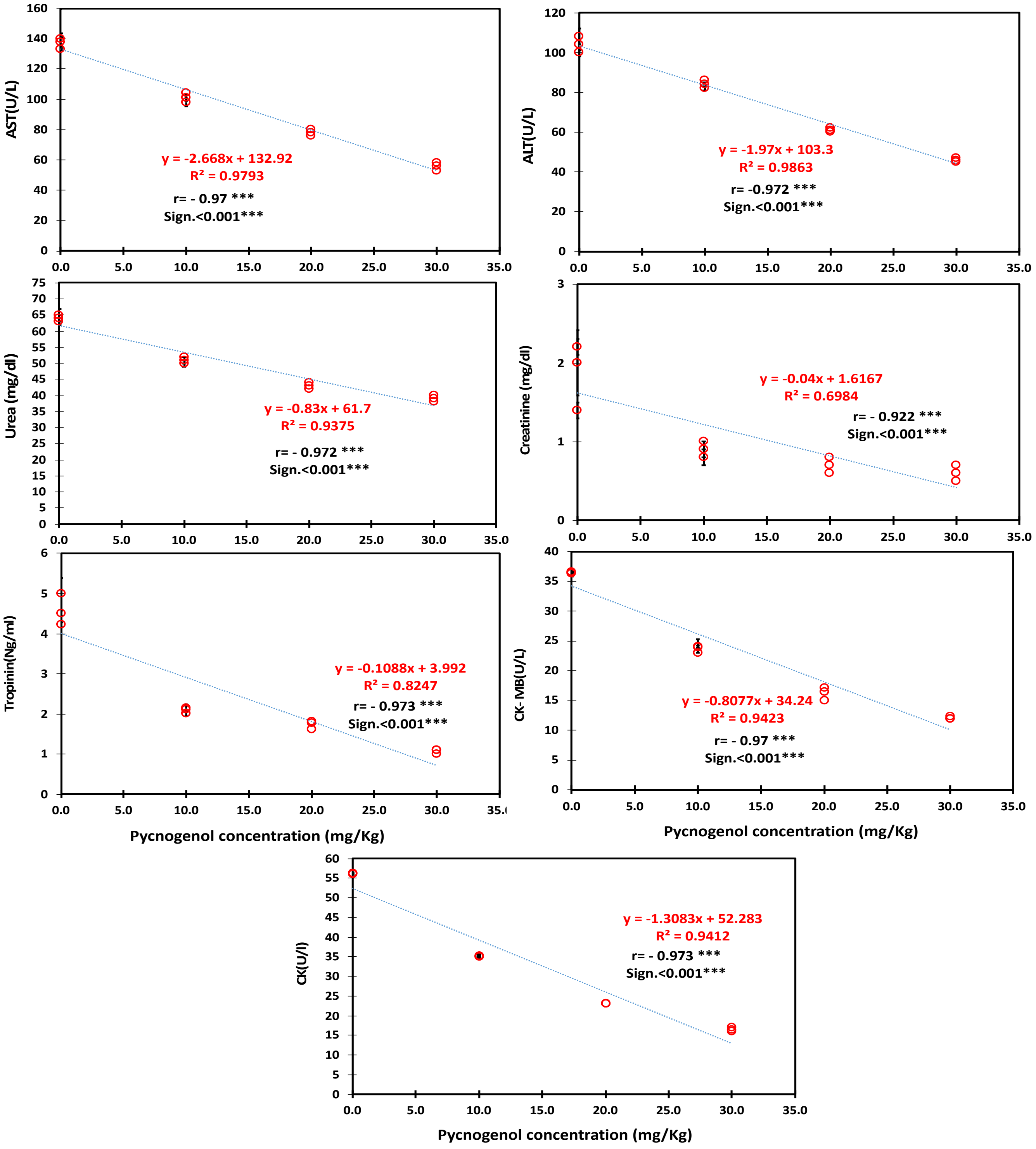

Figure S1. Regression trendline showing the relationship between increasing Pycnogenol levels (10, 20, and 30 mg Kg^{-1}) with various serum biomarkers.

B) Tissue markers: (heart, liver and kidney)

i. Inflammatory biomarkers: NF-κB and TNF-α level

ii. Oxidative stress markers: MDA, ATP and GSH levels, activities of SOD and CAT enzymes

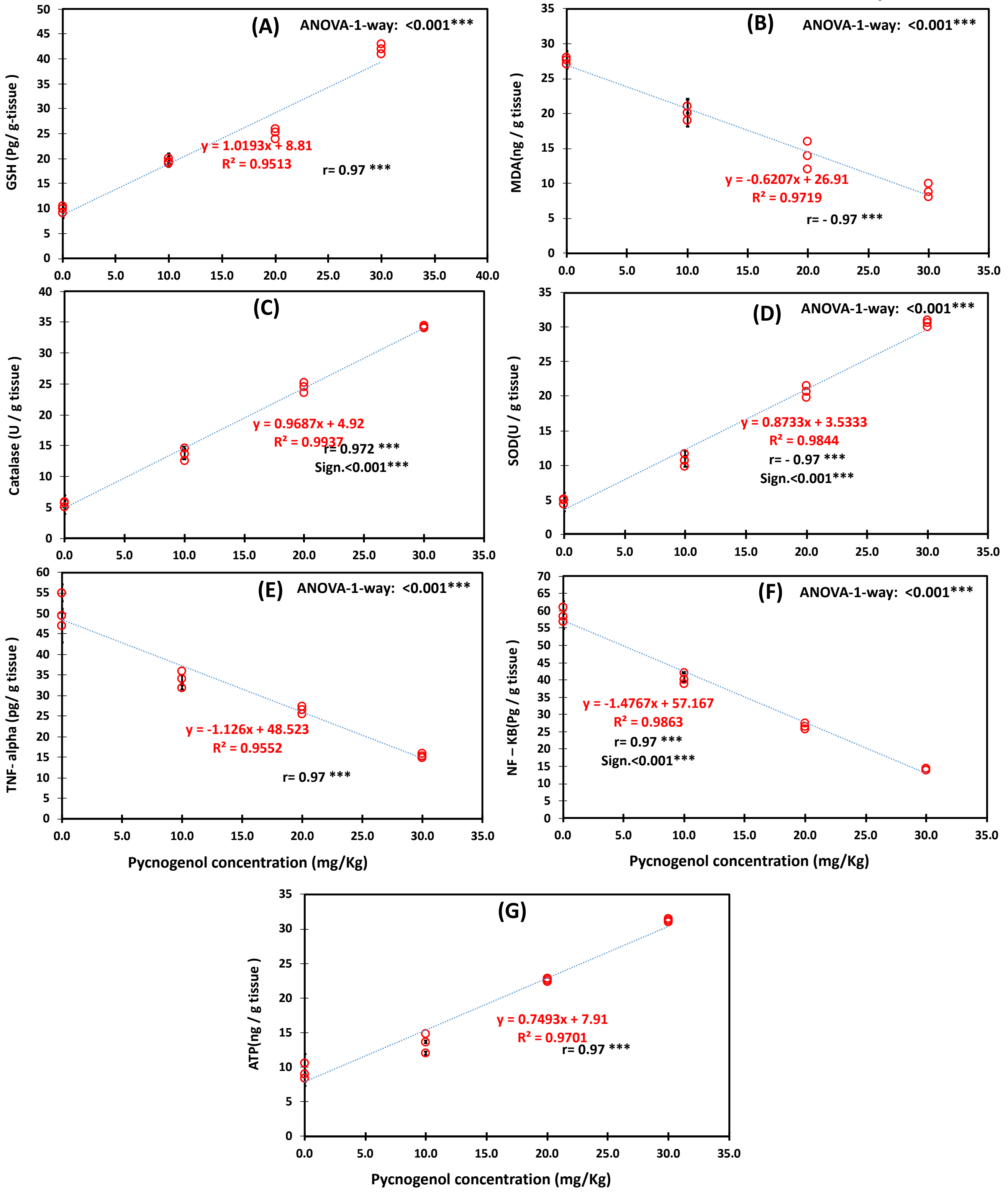

Figure S2. Regression trendline showing the relationship between increasing Pycnogenol levels (10, 20, and 30 mg Kg-1) and various heart biomarkers.

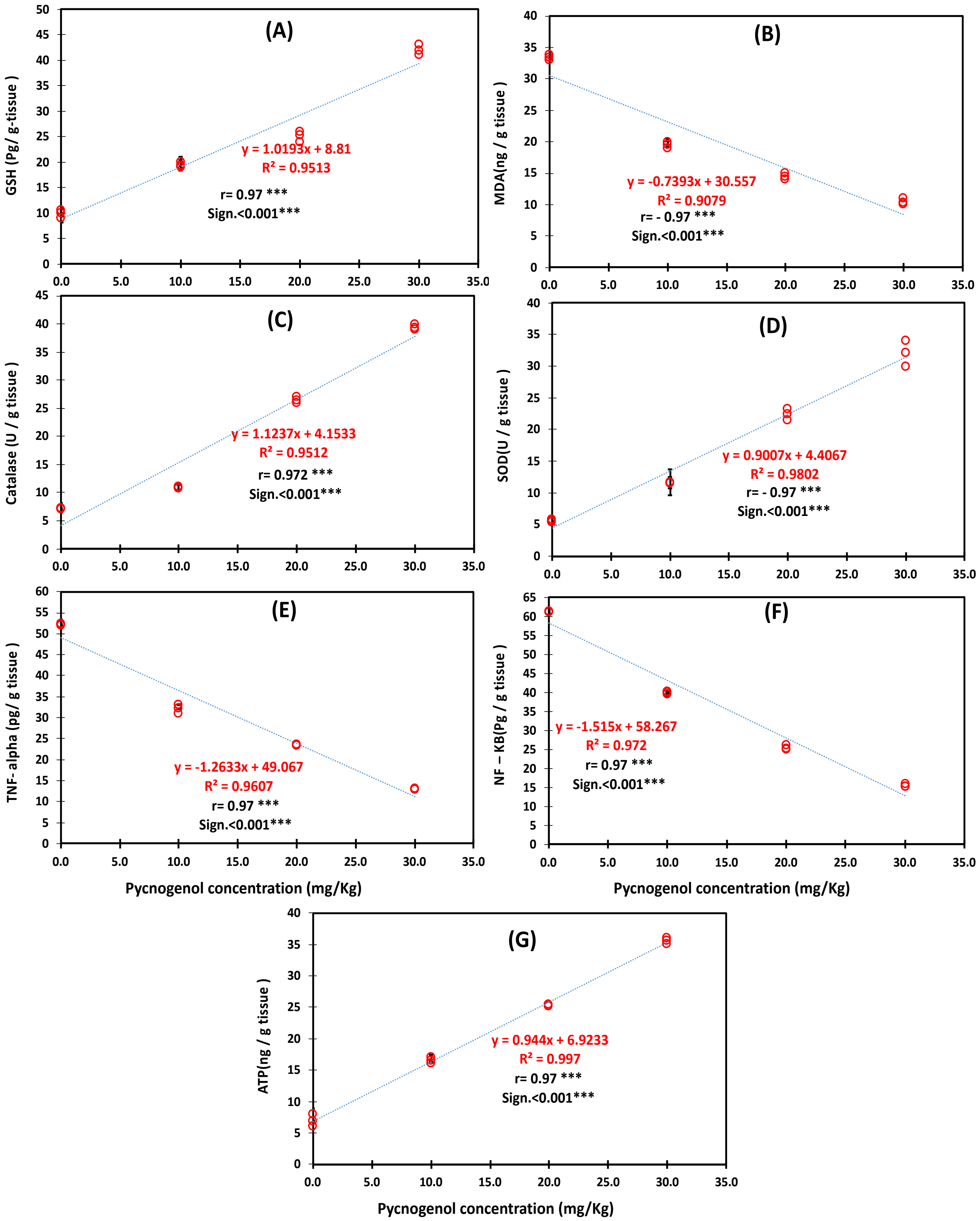

Figure S3. Regression trendline showing the relationship between increasing Pycnogenol levels (10, 20, and 30 mg Kg-1) and various liver biomarkers.

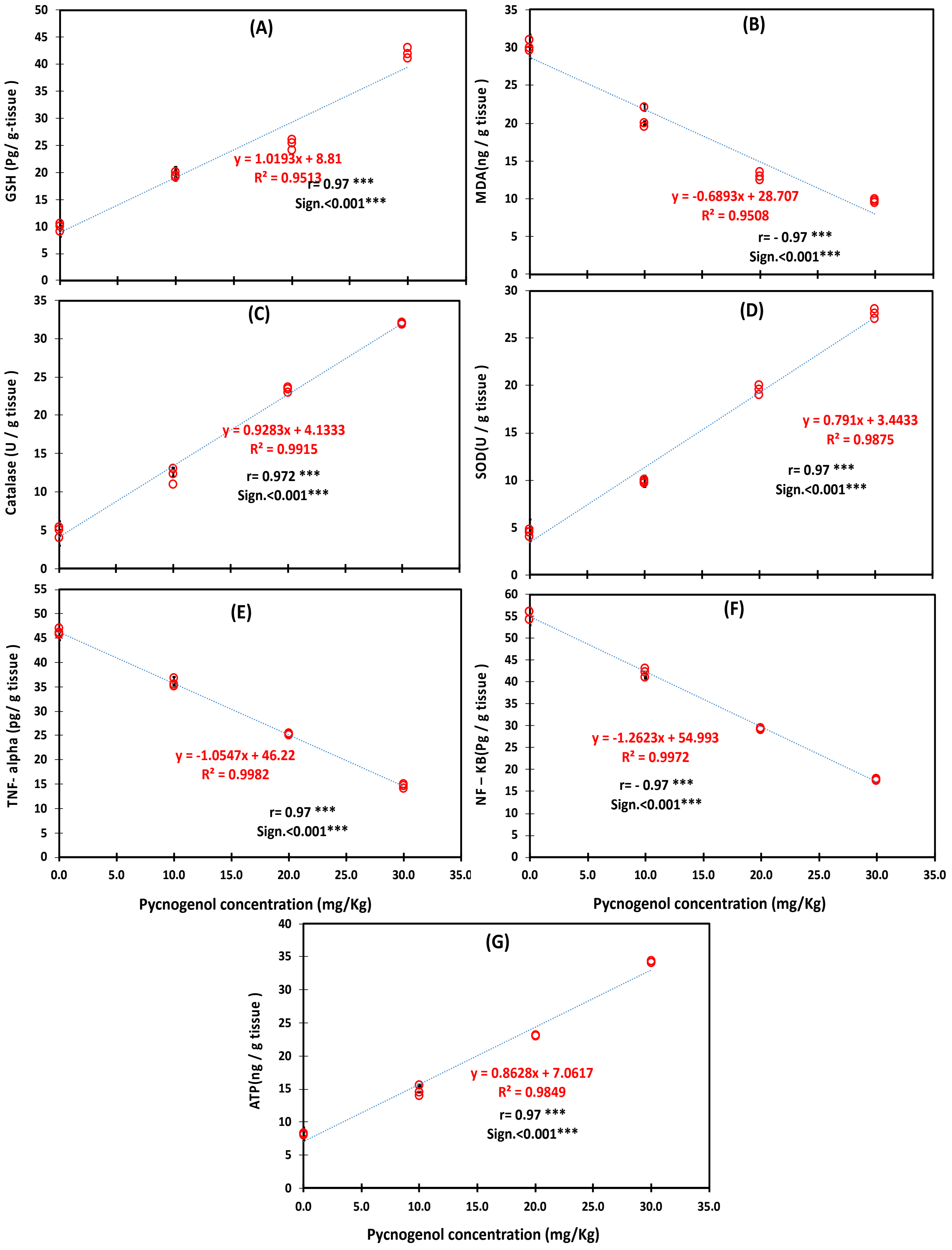

Figure S4. Regression trendline showing the relationship between increasing Pycnogenol levels (10, 20, and 30 mg Kg-1) and various kidney biomarkers.
